# Importance of mediastinal screening-based observation during endoscopic ultrasound to examine gastrointestinal pathologies

**DOI:** 10.1007/s12328-018-0905-x

**Published:** 2018-09-12

**Authors:** Naohito Uchida, Yuko Bando, Sae Hamaya, Yukiko Koyama, Kazuhiro Kozuka, Rie Kawakita, Yuki Sawada, Akihiko Tatemoto, Toshiaki Nakatsu

**Affiliations:** 1 Departments of Gastroenterology, Saint Martin’s Hospital, 1-4-13 Tani-machi, Sakaide City, Kagawa 762-0033 Japan; 2 Departments of Surgery, Saint Martin’s Hospital, 1-4-13 Tani-machi, Sakaide City, Kagawa 762-0033 Japan

**Keywords:** Endoscopic ultrasound (EUS), Screening, Mediastinum, Cardiovascular disease, Myxoma

## Abstract

In all endoscopic ultrasound (EUS) examinations performed at our hospital, the heart, vasculature, and mediastinal lymph nodes from the esophagus are observed after checking for gastrointestinal pathologies. Since the introduction of EUS using a convex linear-array echoendoscope at our hospital in April 2015, EUS examinations have been performed in 371 cases for examining pancreaticobiliary diseases, submucosal tumors, and other pathologies during the 3-year period, till March 2018. We diagnosed 2 patients with asymptomatic cardiovascular disease while observing the mediastinum during EUS examination to examine identified pancreaticobiliary disease. No subjective symptoms associated with cardiovascular disease were observed and the respective conditions had not been identified previously in either case. One case involved a left atrial myxoma while the other involved a saccular aortic aneurysm in the thoracic aorta. A left atrial tumor resection and aortic replacement surgery were performed in each case. Their postoperative courses have been favorable. As cardiovascular diseases are often life-threatening, as in the present 2 cases, observational screening of the cardiovascular system from the esophagus should also be performed during EUS examinations just as the pharyngeal region is examined during upper gastrointestinal endoscopy.

## Introduction

Endoscopic ultrasound (EUS) is useful in the diagnosis of not only pancreaticobiliary and submucosal tumors, but also mediastinal diseases, such as mediastinal tumors and mediastinal lymph node enlargement [1, 2]. We diagnosed 2 patients with asymptomatic cardiovascular disease while observing the mediastinum during EUS examination to examine identified pancreaticobiliary disease. One case involved a left atrial myxoma while the other involved a saccular aortic aneurysm in the thoracic aorta. There were no subjective symptoms associated with these cardiovascular pathologies in either case, and the pathologies had not been detected previously. These cases suggest the importance of observational cardiovascular screening from the esophagus even in cases where EUS is performed to closely examine gastrointestinal disease.

## Case reports

In all EUS examinations performed at our hospital, the heart, vasculature, and mediastinal lymph nodes from the esophagus are observed after checking for gastrointestinal pathologies. Since the introduction of EUS at our hospital in April 2015, EUS examinations have been performed in 371 cases for examining pancreaticobiliary diseases, submucosal tumors, and other pathologies during the 3-year period, till March 2018. The results of these 371 EUS examinations included 1 case of left atrial myxoma and 1 case of saccular aortic aneurysm of the thoracic aorta. No subjective symptoms associated with cardiovascular disease were observed in either case, and the respective conditions had not been identified previously. A convex linear-array echoendoscope (GF-UGT260; Olympus Optical Corp., Tokyo, Japan) connected to an ultrasonography platform (EU-ME2; Olympus Optical Corp., Tokyo, Japan) was used in this study. All EUS examinations were carried out under conscious sedation with i.v. midazolam by an experienced endosonographer (NU) who had 15 years of experience carrying out the procedure.

**Fig. 1:**
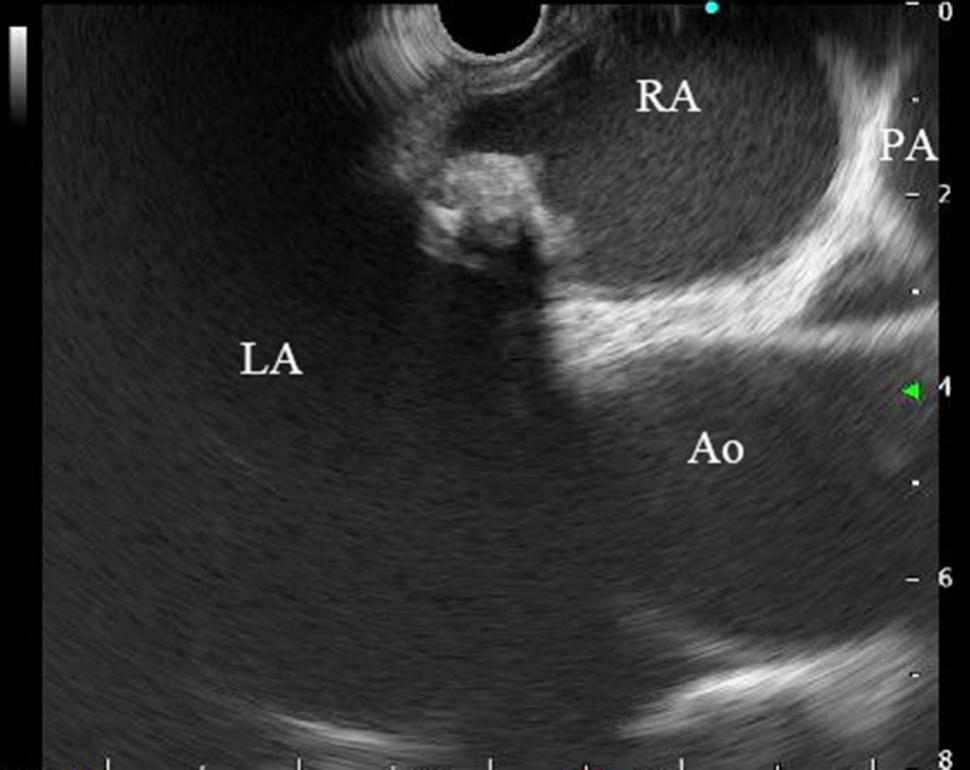
In endoscopic ultrasound, observation of the mediastinum revealed a lesion measuring 15 mm × 9 mm with heterogeneous echoic pattern and partly calcified pattern at the left atrial wall. *LA* left atrium, *RA* right atrium, *Ao* aorta, *PA* pulmonary artery

**Fig. 2:**
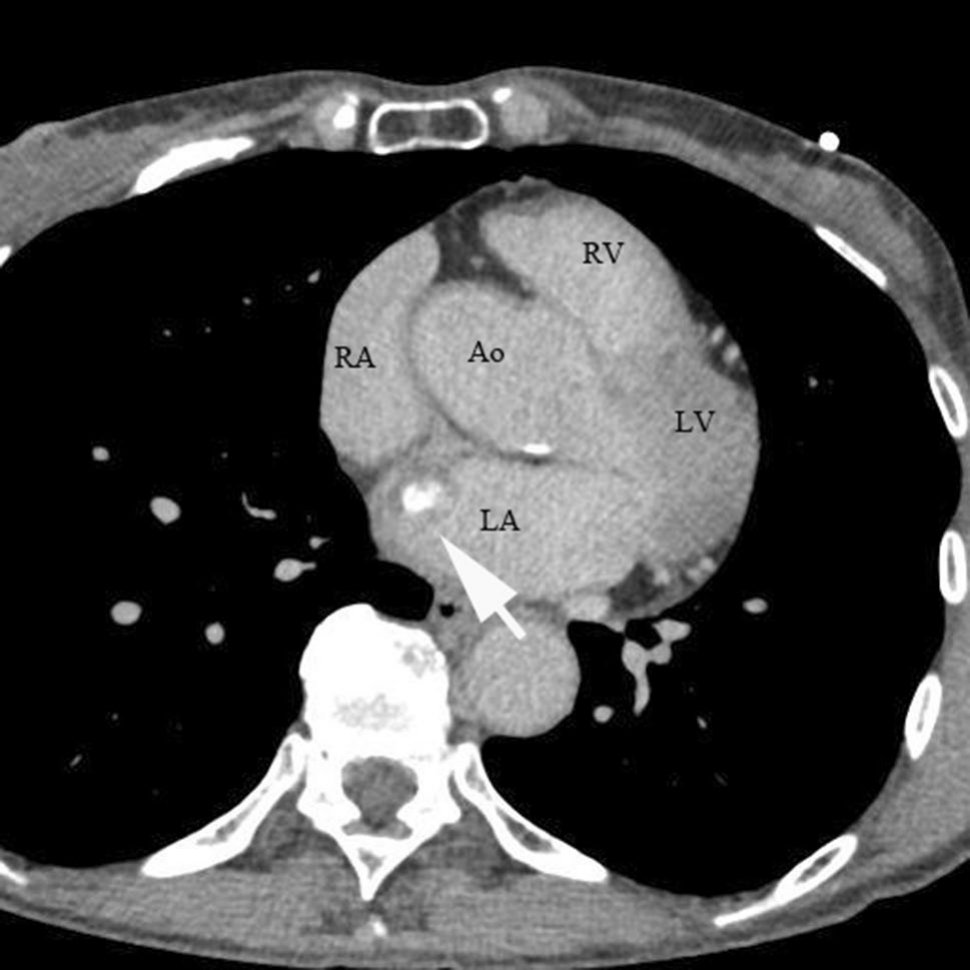
Enhanced computed tomography demonstrated partly calcified tumor at the left atrial wall (arrow). *RA* right atrium, *LA* left atrium, *Ao* aorta, *RV* right ventricle, *LV* left ventricle

**Fig. 3:**
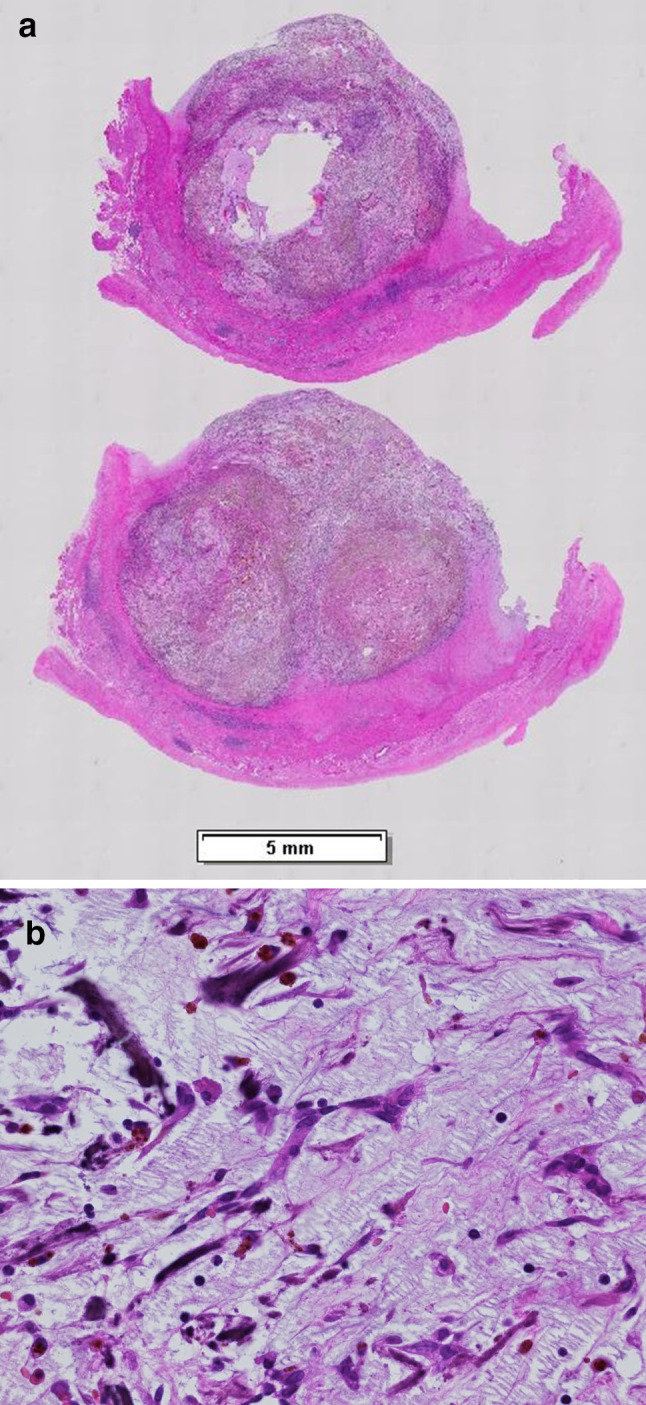
**a** Loupe view of the resected tumor (hematoxylin–eosin stain). **b** Histopathological examination (hematoxylin–eosin stain) revealed small circular and spindle-shaped tumor cells in the mucous stroma, in addition to hemosiderin deposits and linear calcification (Gamna–Gandy bodies)

*Case 1* A 75-year-old woman had been followed-up by our hospital since 2009 via abdominal computed tomographic (CT) examination, magnetic resonance cholangiopancreaticography (MRCP), or other examination methods for intraductal papillary mucinous neoplasm (IPMN). Following its introduction, when EUS was performed as follow-up for IPMN in January 2017, observation of the mediastinum revealed a lesion measuring 15 mm × 9 mm with heterogeneous echoic pattern and partly calcified pattern at the left atrial wall (Fig. 1). A left atrial tumor was suspected based on a contrast CT scan (Fig. 2) and other considerations. A left atrial tumor resection was performed in April 2017. The tumor was a 20 mm × 15 mm broad-based tumor attached to the atrial septum. Histopathological examination revealed small circular and spindle-shaped tumor cells in the mucous stroma, in addition to hemosiderin deposits and linear calcification (Gamna–Gandy bodies), which are indicative of myxoma (Fig. 3a, b). Based on these findings, the lesion was determined as left atrial myxoma. The patient has exhibited a favorable postoperative course and continues to be followed-up regularly for IPMN.

**Fig. 4:**
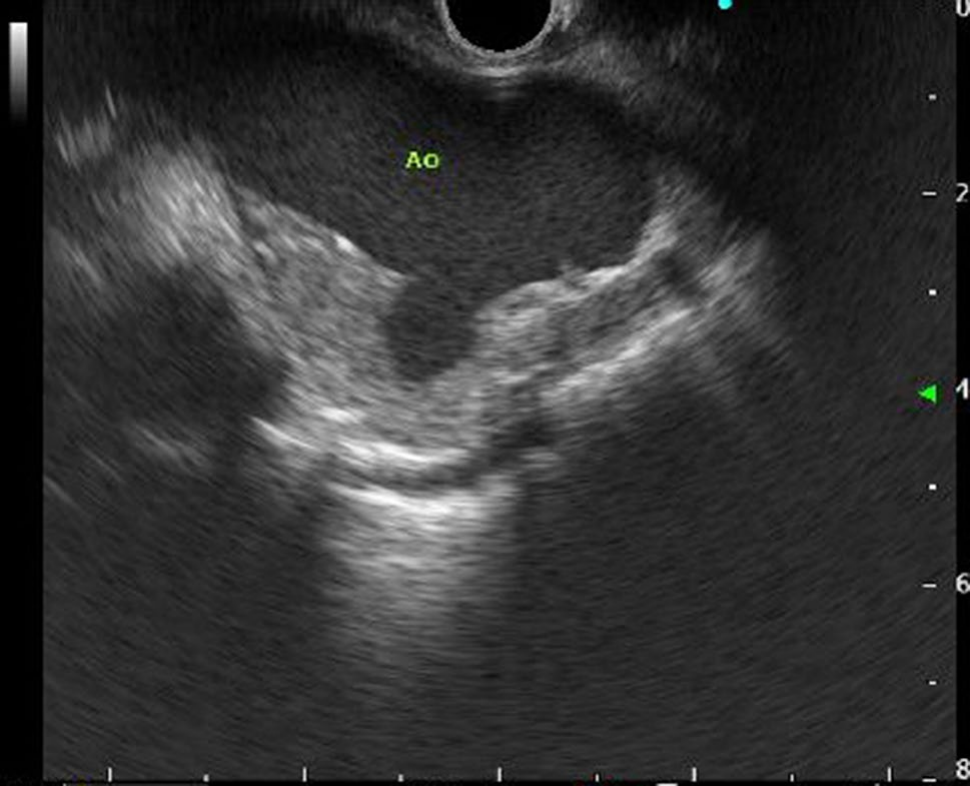
Endoscopic ultrasound from the esophagus revealed aortic wall thickening and a sac-like protrusion of the aortic lumen. *Ao* aorta

**Fig. 5:**
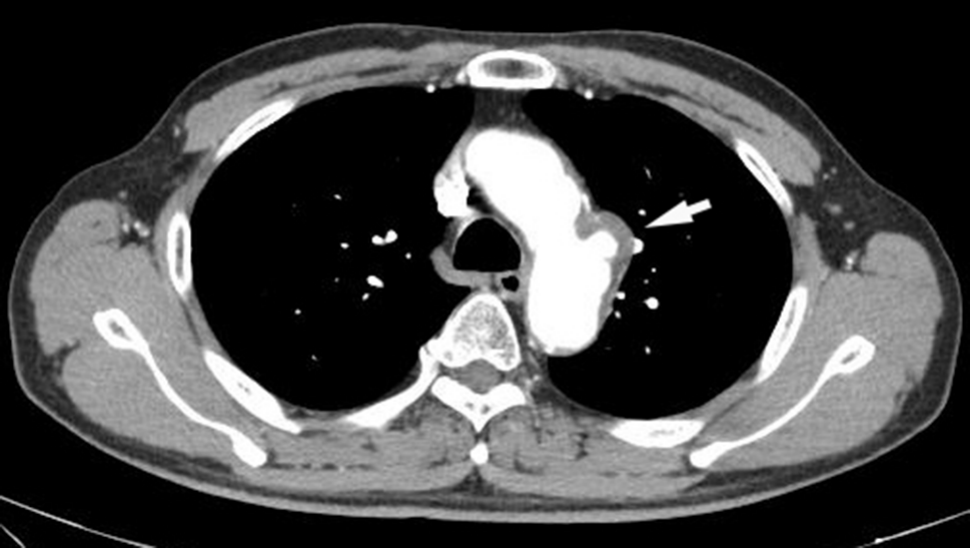
Enhanced computed tomography demonstrated a saccular aneurysm in the aortic arch (arrow)

*Case 2* A 67-year-old man underwent EUS in December 2017 to examine localized gallbladder wall thickening identified at another hospital. Observation from the stomach and duodenum revealed localized wall thickening with Rokitansky–Aschoff sinuses at the base of the gallbladder. This was diagnosed as localized fundal type adenomyomatosis of the gallbladder. Continued observation of the mediastinum revealed aortic wall thickening and a sac-like protrusion of the aortic lumen, and an aortic aneurysm was suspected (Fig. 4). Contrast CT examination revealed a saccular aneurysm in the aortic arch (Fig. 5), which was determined to be operable. Aortic replacement surgery at the aortic arch was performed in March 2018. The patient’s postoperative course has been favorable.

## Discussion

In all EUS examinations performed at our hospital, the mediastinum from the esophagus is observed after checking for gastrointestinal pathologies. The procedure is to first visualize the liver, and subsequently, examine the right atrium from the hepatic vein to the inferior vena cava. Following this, the scope is slightly turned counterclockwise and the left atrium, left ventricle, ascending aorta, pulmonary artery, and the lymph nodes of subcarinal space are observed. Subsequently, the scope is withdrawn and rotated leftward at the site where air was visible through the carina. The aortopulmonary window lymph nodes, aortic arch, and pulmonary artery are then examined. It is important to operate the scope gently during mediastinal observation because the esophagus is limited space compared with the stomach.

Primary cardiac tumor is a rare disease identified in 0.001–0.28% of all autopsies. Approximately, 70% of these are benign, while 30% are malignant. Myxoma is the most common pathology among benign cardiac tumors, commonly occurring in the left atrium. Symptoms include shortness of breath, fainting, and dizziness, which arise due to embolism or hemodynamic disruption caused by mitral valve blockage. There is also a possibility of sudden death if a tumor is affixed to the mitral valve. Echocardiography is useful for diagnosing this condition, in which echogenic luminosity is uneven and sometimes accompanied by calcification. Generally, surgical resection is performed regardless of the tumor size. On the other hands, although aneurysm of the thoracic aorta can cause symptoms such as hoarseness, bloody sputum, and back pain, the majority of cases are asymptomatic. In many cases, the condition is diagnosed by simple chest X-rays or CT examinations for other diseases, with CT examinations being most useful for diagnostic purposes. Once a thoracic aortic aneurysm ruptures, the patient faces a high mortality rate, and surgical treatment must be considered based on the shape of the lesion, size of the aortic aneurysm, rate of expansion, observed symptoms, and other factors. Since saccular aneurysms and pseudoaneurysms are at a higher risk of rupture, surgery is indicated for these conditions. The present case involved a saccular aneurysm, and accordingly surgery was performed as this was the indicated treatment method.

During a literature review, there have been several reports [3–7] regarding incidental diagnosis of other diseases than the gastrointestinal one. For example, the report by Zalts et al. [3] involved a giant left atrial myxoma discovered unintentionally during examination of a gastric submucosal tumor. The small number of such reports regarding incidentally discovered cardiovascular diseases is probably because of few institutions where mediastinal observation during EUS examinations are actively performed. The number of EUS examinations performed is likely to increase in the future and the number of unintentionally discovered cases of cardiovascular disease during EUS examination is also likely to increase. Two cases out of 317 EUS examinations in our report suggest the importance of observational cardiovascular screening from the esophagus even in cases where EUS is performed to closely examine gastrointestinal disease. Screening of the cardiovascular and mediastinal lymph nodes from the esophagus requires only 1 min approximately. As cardiovascular diseases are often life-threatening, as in the present 2 cases, observational screening of the cardiovascular system from the esophagus should also be performed during EUS examinations just as the pharyngeal region is examined during upper gastrointestinal endoscopy.
